# An Optimized Langendorff-free Method for Isolation and Characterization of Primary Adult Cardiomyocytes

**DOI:** 10.21203/rs.3.rs-4131724/v1

**Published:** 2024-03-29

**Authors:** Azadeh Nikouee, John Q. Yap, David J. Rademacher, Matthew Kim, Qun Sophia Zang

**Affiliations:** Loyola University Chicago

## Abstract

Isolation of adult mouse cardiomyocytes is an essential technique for advancing our understanding of cardiac physiology and pathology, and for developing therapeutic strategies to improve cardiac health. Traditionally, cardiomyocytes are isolated from adult mouse hearts using the Langendorff perfusion method in which the heart is excised, cannulated, and retrogradely perfused through the aorta. While this method is highly effective for isolating cardiomyocytes, it requires specialized equipment and technical expertise. To address the challenges of the Langendorff perfusion method, researchers have developed a Langendorff-free technique for isolating cardiomyocytes. This Langendorff-free technique involves anterograde perfusion through the coronary vasculature by clamping the aorta and intraventricular injection. This method simplifies the experimental setup by eliminating the need for specialized equipment and cannulation of the heart. Here, we introduce an updated Langendorff-free method for isolating adult mice cardiomyocytes that builds on the Langendorff-free protocols developed previously. In this method, the aorta is clamped *in situ*, and the heart is perfused using a peristaltic pump, water bath, and an injection needle. This simplicity makes cardiomyocyte isolation more accessible for researchers who are new to cardiomyocyte isolation or are working with limited resources. In this report, we provide a step-by-step description of our optimized protocol. In addition, we present example studies of analyzing mitochondrial structural and functional characteristics in isolated cardiomyocytes treated with and without the acute inflammatory stimuli lipopolysaccharide (LPS).

## 1. Introduction

Isolation of cardiomyocytes from adult mice is a commonly used technique in cardiovascular research, facilitating the mechanistic investigation of cardiac physiology and heart diseases at the cellular level. The traditional method of isolating primary cardiomyocytes utilizes the technique of retrograde heart perfusion, known as the Langendorff heart perfusion model. This method was developed by Dr. Oscar Langendorff and his colleagues at the University of Rostock in Germany towards the end of the 19th century (1). The procedure involves excising the heart, inserting a cannula into the aorta, and perfusing the heart retrogradely with an oxygenated digestion solution through a specialized apparatus. In this setup, continuous heart beating is maintained by perfusing the heart with oxygenated buffer solutions. This foundational work laid the groundwork for significant advancement in studying cardiac physiology and pathology under various conditions. Over the years, modifications and refinements to the Langendorff technique have been made to improve the isolation of cardiomyocytes from perfused hearts across various species, especially rodents (2, 3). A major advancement came with the introduction of enzymatic digestion methods combined with Langendorff perfusion in the 20th century, utilizing enzymes such as collagenase to dissociate cardiac tissue into individual cells while maintaining cell viability and functionality (4).

While the Langendorff method of isolation has been instrumental in advancing cardiac research, it poses technical challenges due to its intricate nature and necessitates the use of specialized equipment. To overcome these challenges, recent innovations have led to the development of a Langendorff-free method for isolating adult mouse cardiomyocytes, achieving comparable yields to the traditional method (5, 6). This approach involves clamping the aorta, excising the heart, placing it in a petri dish with a warm enzymatic digestion solution, and anterograde perfusion by left ventricular injection. The primary benefits of this method include the elimination of aortic cannulation and the reduced need for specialized equipment.

Based on published protocols (3, 5, 7), we have optimized the procedure of cardiomyocyte isolation from adult mice to increase ease of handling and reproducibility of the results. This report details our protocol modifications and potential downstream applications, as described in the Method section. In the results presented herein, we focused on characterizing mitochondrial structure and function in isolated adult cardiomyocytes, reflecting our group’s research emphasis (8–10). Given that the heart has a significantly higher energy demand compared to other organs, and cardiomyocytes rely on functional mitochondria for energy production, mitochondrial health is a critical indicator of cardiomyocyte vitality (11). The results presented herein show that our optimized protocol yields viable cardiomyocytes with preserved morphology and functionality, thus offering a valuable tool for investigating heart physiology and pathology.

## 2. Results

### 2.1 A simplified protocol of isolating primary cardiomyocytes from adult mice.

**Figure 1:**
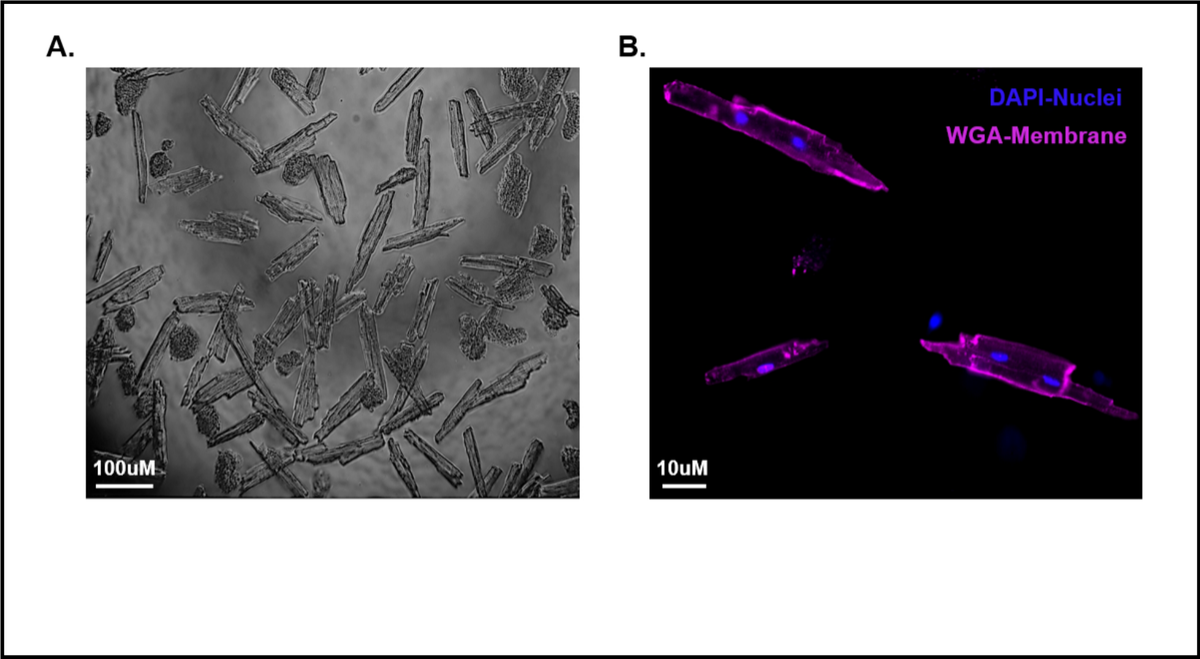
Cardiomyocytes post-isolation. **A**) Brightfield image of isolated cardiomyocytes after plating. **B)** Confocal imaging of isolated cardiomyocytes stained with the cell membrane marker wheat germ agglutinin (WGA) (colored purple) and the nuclei marker DAPI (colored blue).

By using our simplified cardiomyocyte isolation protocol described in the Method section, we consistently achieve yields comparable to those obtained through the traditional Langendorff methods (Fig. 1). The resulting cardiomyocytes exhibit the characteristic log-like shape, with intact plasma membranes and well-preserved t-tubules, as shown in Figs. 1A and 1B.

### 2.2. Transmission electron microscopy analysis of mitochondrial structure, lipid droplets, and autophagic response in isolated cardiomyocytes

**Figure 2:**
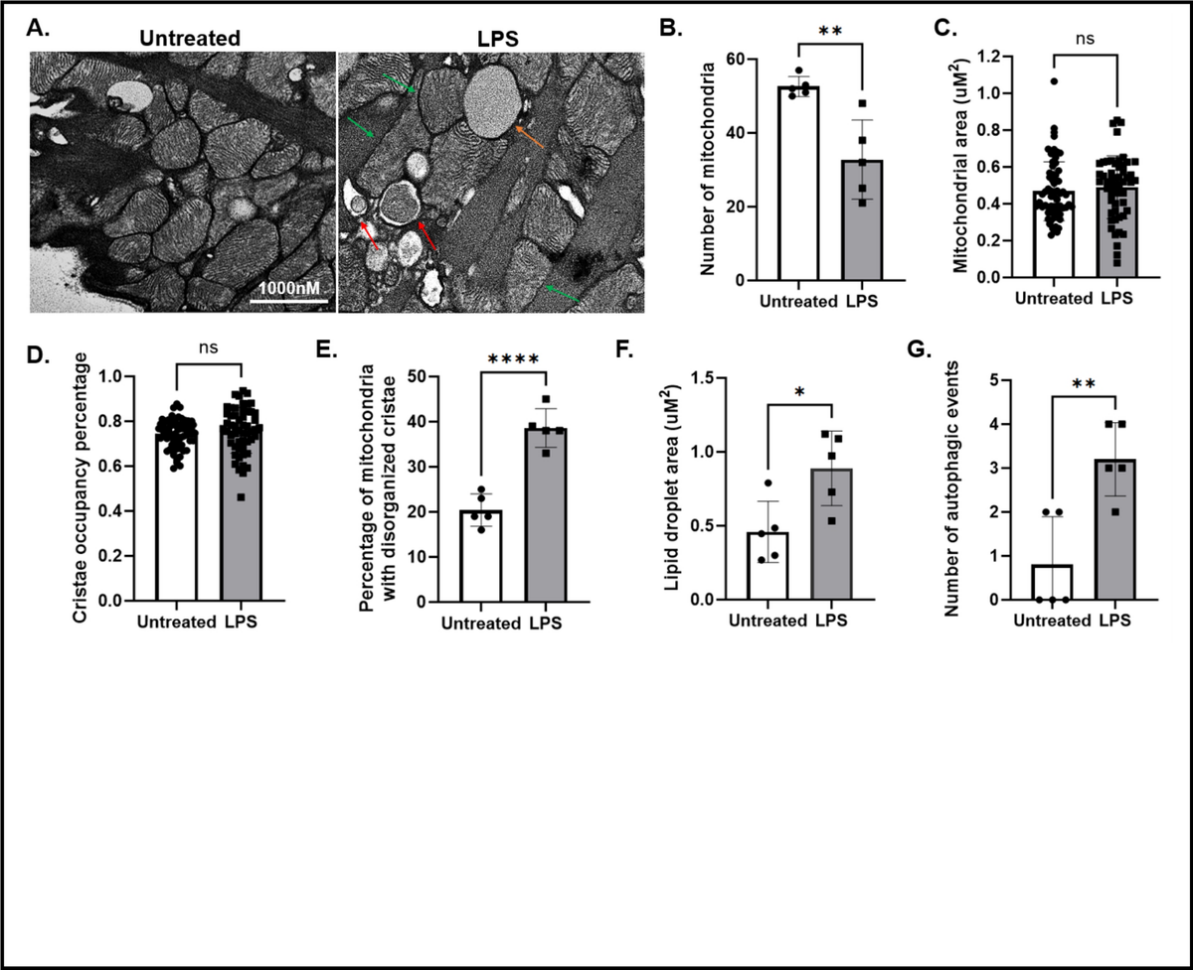
Analysis of mitochondria morphology, lipid droplet accumulation and autophagy in isolated cardiomyocytes using TEM. Cardiomyocytes were isolated from male adult mice that were treated with or without LPS (5mg/kg) for 18 hours post injection. **A**) TEM images of cardiomyocytes untreated and treated with LPS. Red arrows show autophagic events, orange arrow shows a lipid droplet, and green arrows show mitochondria with disorganized cristae. Images are prepresentative for *n* = 2 mice/group. **B)** Number of mitochondria per 50uM area **C)** Mitochondrial area per 50uM area. **D**) Percentage of cristae occupancy relative to total mitochondria area **E**) Percentage of mitochondria with disorganized cristae relative to the number of mitochondria per 50uM area. **F**) Lipid droplet area per 50uM area **G**) number of autophagic events per per 50uM area. In **B-G**, values were expressed as mean ± SD and analyzed by a one-way *t*-test. Significance was determined by a p value <.05 and is shown by astericks.

Transmission electron microscopy (TEM) is a powerful imaging modality for assessing organelle structures at high resolution. Previously, our group used TEM to show that mice intraperitoneally injected with LPS displayed increased disruption of mitochondria cristae structure in cardiac tissue slices (10). In this report, we examined whether an acute inflammatory challenge by LPS alters mitochondria morphology in cardiomyocytes isolated from wild type adult male mice. We have optimized the procedure of preparing cardiomyocyte samples for TEM imaging based on previously published studies (12–14). Experimental details are provided in the Method section. The isolated cardiomyocytes contain mitochondria with intact cristae, having consistent and uniform morphology throughout the mitochondrial network (Fig. 2A), suggesting that the mitochondria maintain a healthy physiological status post-isolation. When treated with LPS, the cells presented a significant reduction in the number of mitochondria (Fig. 2B), despite no significant alterations in the total mitochondrial area (Fig. 2C) or cristae occupancy percentage (Fig. 2D). Additionally, a significant increase in the percentage of mitochondria exhibiting disorganized cristae in response to LPS was detected (Fig. 2E), consistent with our previous published results demonstrating sepsis-induced mitochondrial damage in the heart (10, 15). Furthermore, we observed a significant increase in the area occupied by lipid droplets in response to the LPS challenge (Fig. 2F), suggesting that the LPS challenge may cause an accumulation of fatty acids stored in lipid droplets as metabolic substrate supply. However, this hypothesis will need further evaluation in future studies. We also detected an LPS-induced increase in autophagy in the cardiomyocytes, signified by the formation of autophagosomes (Fig. 2G).

### 2.3. Measurement of mitochondrial membrane potential in isolated cardiomyocytes.

**Figure 3:**
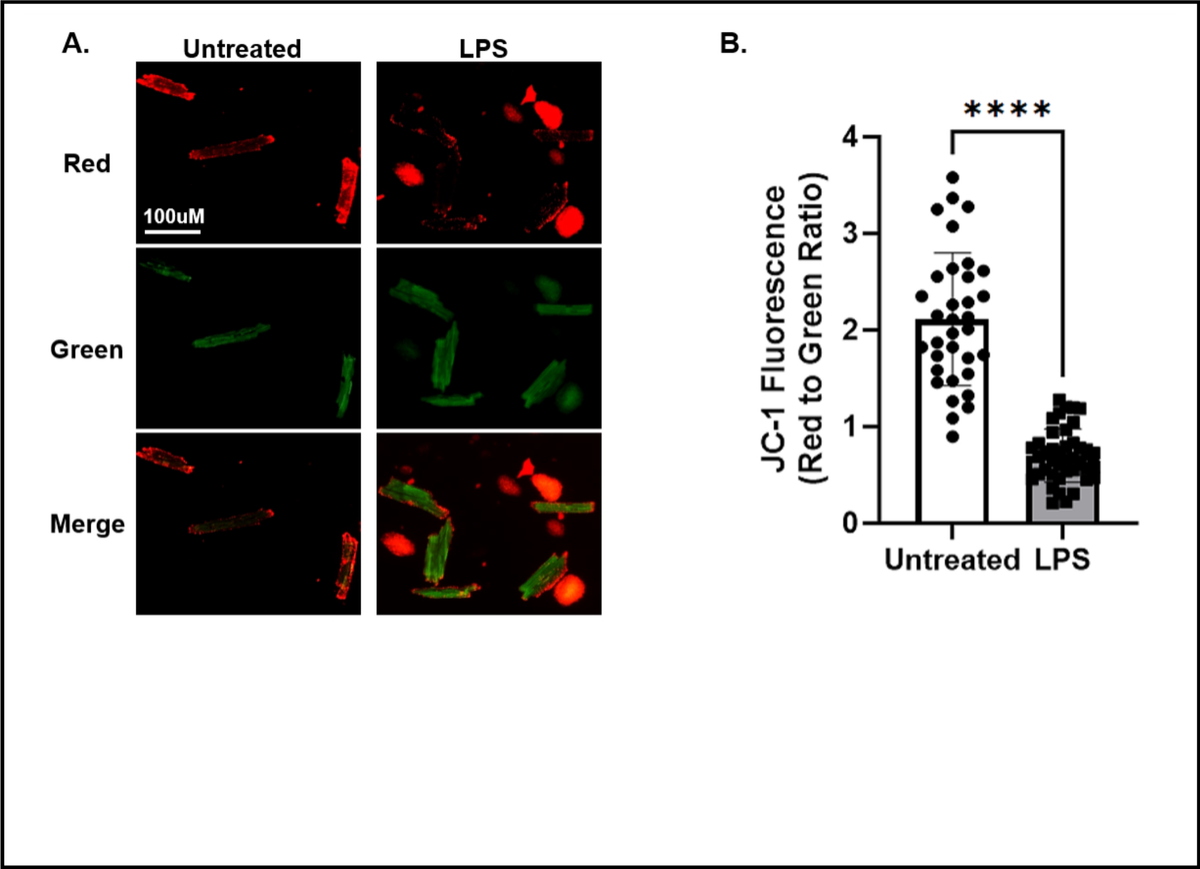
Assessment of mitochondrial membrane potential in isolated cardiomyocytes. **A)**confocal imaging of JC-1 in in untreated and LPS treated cardiomyocytes. The scale bar in the upper left panel equals 100 μm and is valid for all panels. Images are representative of 30–50 cardiomyocytes analyzed from n=2 mice/group. **B)** Quantification of JC-1 fluorescence in untreated and LPS treated cardiomyocytes. Values were expressed as mean ± SD and analyzed by a one-way *t*-test. Significance was determined by a p value <.05 and is shown by astericks.

To evaluate mitochondrial function in isolated cardiomyocytes, we chose to assess mitochondrial membrane potential, a crucial indicator for mitochondrial function and physiological fitness. To do this, isolated cardiomyocytes were incubated with JC-1 dye (Fig. 3A), which changes fluorescent properties based on the mitochondrial membrane potential. At low mitochondrial membrane potential, JC-1 takes on a monomeric form and emits a green fluorescence when excited. However, at higher mitochondrial membrane potential, JC-1 molecules aggregate in the mitochondria and emit a red fluorescence when excited. A lower red-to-green ratio suggests that there is increased mitochondrial depolarization and mitochondrial dysfunction. In untreated isolated cardiomyocytes, we observed a high red-to-green ratio reflecting mitochondrial membrane potential, indicating healthy cardiomyocytes with properly functioning mitochondria (Fig. 3B). However, LPS treatment significantly decreased the membrane potential by more than 50%, suggesting that the mitochondrial function has been compromised in the cardiomyocytes (Fig. 3B).

### 2.4. Quantifying reactive oxygen species production in isolated cardiomyocytes.

**Figure 4:**
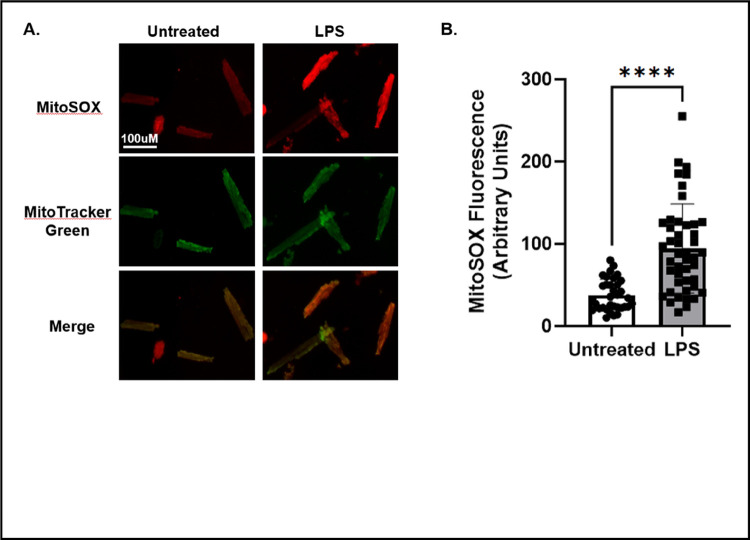
Analysis of ROS production in isolated cardiomyocytes. **A)**Confocal imaging of MitoSOX Red and MitoTracker Green in untreated and LPS treated cardiomyocytes. The scale bar in the upper left panel equals 100 μm and is valid for all panels. Images are representative of 30–50 cardiomyocytes analyzed from n=2 mice/group. **B)** Quantification of MitoSOX Red fluorescence in untreated and LPS treated cardiomyocytes. Images are representative of 30–50 cardiomyocytes analyzed from n=2 mice/group. Values were expressed as mean ± SD and analyzed by a one-way *t*-test. Significance was determined by a p value <.05 and is shown by astericks.

In healthy cardiomyocytes, low levels of reactive oxygen species (ROS) are generated when electrons that are leaked through the electron transport chain (ETC) interact with oxygen molecules to form superoxide radicals (16). However, in unhealthy cardiomyocytes, mitochondrial dysfunction leads to increased leakage of electrons from the electron transport chain, resulting in increased levels of ROS (17). It has been shown that treatment of cardiomyocytes with low concentrations of LPS increases ROS production (18). To assess ROS production in isolated cardiomyocytes, we incubated cardiomyocytes in MitoSOX Red, a fluorescent dye specific for detecting superoxide production in mitochondria from live cells. Cardiomyocytes were counterstained with the mitochondrial marker, MitoTracker Green (Fig. 4A). Compared to the untreated cardiomyocytes, the fluorescent intensity of MitoSOX Red increased significantly in cardiomyocytes treated with LPS (Fig. 4B). This result is consistent with previous findings showing increased ROS production in cardiomyocytes during acute inflammation (19).

## 3. Discussion

Research in our laboratory has been focused on understanding the complex dynamics of sepsis-induced cardiomyopathy, with a particular interest in how acute inflammatory stimuli from infections impact the mitochondria and mitochondria-associated membranes within the myocardium (9, 10, 20). Previously, we utilized TEM imaging to examine the ultrastructural details of the myocardium in cardiac tissue samples. Despite the valuable insights gained, the entire process, from sample preparation to imaging and subsequent analysis, proved to be both time-consuming and resource-intensive. The experiment requires specialized chemicals and equipment, alongside the expertise of trained personnel. Moreover, the extensive nature of the sample preparation protocol increases the risk of introducing artifacts, thereby impacting the consistency and reliability of experimental outcomes. The procedure of tissue sample preparation starts with whole-heart perfusion, followed by dehydration and embedding steps, in which any tissue shrinkage or distortion could adversely affect the integrity of the ultrastructural information obtained from TEM images. Moreover, heart tissue presents inherent complexities due to its heterogeneous cellular composition, including cardiomyocytes, fibroblasts, and endothelial cells. The presence of specialized structures, such as intercalated discs, along with the varied orientation of heart muscle fibers, further complicates the TEM imaging process and complicates the interpretation of imaging data.

In this report, we developed a method that allows for the simplified, cost-effective, time-efficient, and highly reproducible isolation of viable cardiomyocytes from adult mouse models. This method significantly minimizes the risk of human error during the isolation process. Post-isolation, the cardiomyocytes demonstrated preservation of their physiological properties (Fig. 1).

Subsequent TEM imaging of the isolated cell samples revealed findings that were in alignment with observations from intact heart tissue. As shown in Fig. 2, in addition to observing the ultrastructural morphology of mitochondria, we quantified the number of mitochondria, autophagy levels (autophagosomes and autolysosomes), and lipid droplets. Our analysis indicated that exposure to an acute inflammatory challenge, such as LPS, leads to severe mitochondrial damage characterized by disorganized cristae, reduced mitochondrial numbers, increased lipid droplets, and enhanced autophagic response. TEM analysis at the cellular level allows us to determine the intricate changes within the mitochondrial cristae, the surrounding membrane structures, and their interactions with lipid droplets in cardiomyocytes. We noticed that the lipid droplet features were significantly more evident in these cardiomyocyte images compared to whole heart tissue. This advantage is likely due to the fact that processing TEM samples prepared from isolated cells achieves a higher specificity in staining, thereby enhancing the contrast of ultrastructural components under TEM. Together, these results are consistent with previous findings seen in cardiomyocytes during acute inflammation, in which increased mitochondrial damage triggers activation of the autophagy/mitophagy processes (10), resulting in reduced mitochondrial numbers (10, 21). The decrease in functional mitochondria has an effect on diminishing the capacity for lipid utilization, resulting in an accumulation of lipid droplets (22, 23).

Additionally, live cell-based detection using fluorescent labeling can be successfully applied in the isolated cardiomyocytes, which is another advantage compared to using heart tissue samples. In our study, we used JC-1 and MitoSOX to assess mitochondrial potential and superoxide production, respectively. As shown in Figs. 3 and 4, we obtained results showing that LPS challenge caused a significant decrease in mitochondrial membrane potential and an increase in superoxide production, indicating the development of mitochondrial functional deficiency accompanied by oxidative stress.

In summary, we demonstrate an optimized method for isolating viable cardiomyocytes from adult mouse models. This simplified, straightforward method allows us to overcome the limitations associated with the traditional Langendorff method and sample preparation using cardiac tissue, reducing the time and resource demands, minimizing risk of artifacts, and enhancing our ability to obtain ultrastructural data with more defined details. The presented results we obtained from these isolated cardiomyocytes are consistent with findings obtained from the heart tissue. We anticipate that improving the methodology will help facilitate precise, reproducible, and insightful analysis of cardiomyocyte pathology.

## 4. Method

### 4.1. Experimental animals

All methods are reported in accordance with ARRIVE guidelines 2.0 for the reporting of animal experiments (24). Wild-type (WT) C57BL/6 mice were purchased from The Jackson Laboratory (Bar Harbor, ME) and housed at the in-campus mouse breeding core facility at Loyola University Chicago (LUC). Animals were conditioned in-house for 5–6 days after arrival with commercial diet and tap water available ad *libitum*. For the purposes of this study, male mice aged between 3 and 4 months were selected. All animal-related procedures described in this study underwent a comprehensive review and were conducted under the oversight of the LUC Institutional Animal Care and Use Committee. These procedures adhered to the standards outlined in the National Research Council’s “Guide for the Care and Use of Laboratory Animals” when establishing animal research standards. Primary cardiomyocytes were isolated from the heart tissue according to the procedure described below. In some experiments, cells were treated with LPS (MilliporeSigma, Burlington, MA; catalog number L3012) at the concentrated indicated. all methods are reported in accordance with ARRIVE guidelines (https://arriveguidelines.org) for the reporting of animal experiments.

### 4.2. Solutions made prior to the day of cardiomyocyte isolation

#### 2× base buffer:

To make the solution, add the components listed in Table 1 to 500 ml of distilled water. Gradually adjust the pH to 7.8 by adding NaOH dropwise. Once prepared, this solution can be stored at 4°C for up to 2 weeks.

#### EDTA buffer:

To prepare 250 ml 1× EDTA buffer solution, start by mixing 125 ml of a 2× base buffer with 125 ml of distilled water, and dissolve 365.3 mg of EDTA disodium dihydrate (Sigma, E5134) into this 1× base buffer solution. Adjust the pH to 7.8 by adding NaOH dropwise, and filter the solution through a 0.2 μm filter to ensure sterility. This solution can be stored at 4°C for up to 2 weeks. For each experiment, approximately 50 mL of this buffer will be required.

#### Perfusion buffer:

To prepare 750 ml 1× perfusion buffer, start by mixing 375 ml of a 2× base buffer with 375 ml of distilled water, and dissolve 71.4 mg of MgCl (Sigma, M8266) into this 1× base buffer. Adjust the pH to 7.8 by adding NaOH dropwise, and filter the solution through a 0.2 μm filter to ensure its sterility. This prepared solution can be stored at 4°C for up to 2 weeks.

#### Enzymes:

Dilute 1000 mg of collagenase 2 (Worthington, LS004176), collagenase 4 (Worthington, LS004188), and protease XIV (Sigma Aldrich, P5147) separately in 20 ml of distilled water. Each enzyme should be prepared one at a time on ice and aliquoted into volumes of 525 μL for collagenase 2 and collagenase 4 and 55 μL for protease XIV. Aliquots can be stored at −80°C for up to 6 months.

### 4.3. Solutions made on the day of cardiomyocyte isolation

#### Digestion buffer:

Digestion buffer can be prepared by mixing the gradients listed in Table 2.

#### Stop buffer:

Add 1 ml of fetal bovine serum (FBS) to 19 ml of perfusion buffer, and let the solution equilibrate to room temperature before use.

#### Plating media:

Plating media can be prepared by mixing the components listed in Table 3. The total volume of plating media required will vary based on the quantity of downstream experiments planned. Prior to use, allow the solution to equilibrate to room temperature.

#### Calcium re-introduction solutions:

Calcium re-introduction solutions can be prepared by mixing the components in Table 4. Prior to use, allow the solution to equilibrate to room temperature.

#### Tyrode buffer:

Dissolve 35.28 mg of CaCl2·6H2O (Sigma, 21108) in 200 mL of perfusion buffer, and pass the solution through a 0.2 μm filter for sterilization. This solution can be stored at 4°C for up to 2 weeks.

### 4.4. Preparation of cell culture plates

Coat 35 mm plates with 0.1 mg/mL poly-D-Lysine (PDL) (Sigma, 6407) for 30 minutes at room temperature. Then, aspirate the PDL solution and allow the plates to dry for at least 30 minutes in a 37°C incubator. Prior to cell plating, rinse the plates once with plating media.

### 4.5. Preparation of surgical equipment and area

#### Tubing preparation:

One day prior to the experiment, flush the peristaltic pump tubing (Harvard Apparatus, 72–0668) with 70% ethanol followed by water to ensure cleanliness. Allow the tubing to air dry completely.

#### Surgical area preparation:

**Figure 5:**
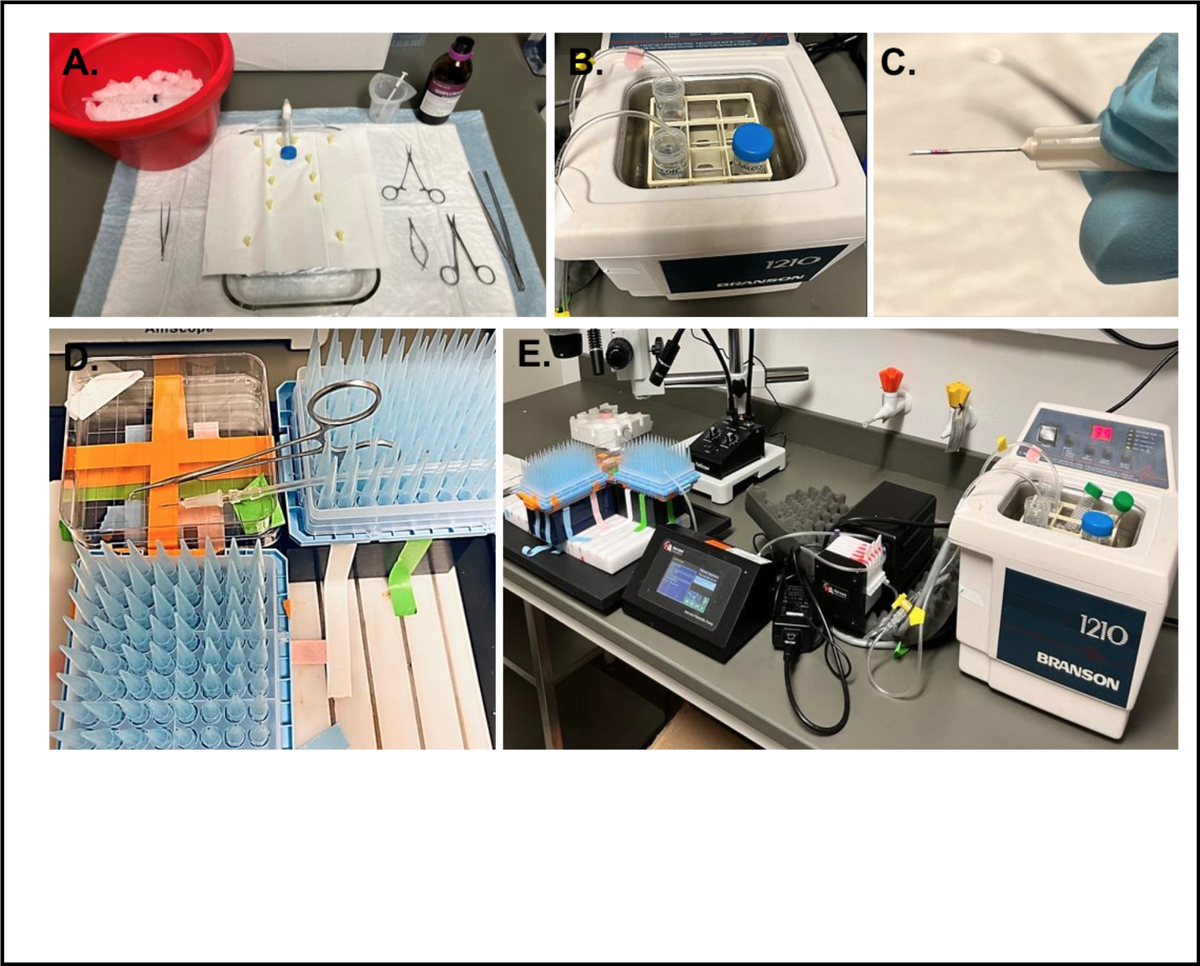
Setup for cardiomyocyte isolation. **A)**Surgical tools needed for heart excision, including a surgical table, a 5 ml syringe containing ice-cold EDTA buffer, tweezers, sharp-tipped scissors, Vannas scissors, and curved-ended Reynolds hemostatic forceps. **B)** EDTA and digestion buffers in a 37°C water bath for preparation. **C)**Injection needles marked with nail polish to ensure penetration depth of only 3mm into the heart. **D)** A custom-made holder securely positions the injection needle and tubing. **E)**An overview of the complete perfusion system.

Clean the surgical area thoroughly with 70% ethanol before the procedure. Sterilize all surgical instruments as shown in Figure 5A to maintain aseptic conditions.

#### Buffer preparation:

- On the experiment day, prepare three sterile 50 ml conical tubes: one with 50 ml of EDTA solution and the other two with 50 ml of digestion buffer each. The second digestion buffer conical serves as a refill for the one connected to the pump.
- Place both the EDTA and digestion buffer solutions in a 37°C water bath to reach the desired temperature. Once at 37°C, introduce the peristaltic pump tubing into these solutions (Figure 5B).

#### Pump and needle preparation:

- Set the peristaltic pump (Harvard Apparatus, 70–7000) to 30ml/min and allow approximately 10 ml of digestion buffer to circulate through the tubing to expel any air bubbles. Repeat the process with the EDTA buffer.
- To prevent the injection needles from being inserted too deeply, mark two 27G needles at a distance of 3 mm from the needle tip using colored nail polish (Figure 5C).

#### Syringe and needle setup for ventricular injection:

- Fill a sterile 5 ml syringe with 5 ml of cold EDTA buffer and attach one of the marked 27G needles. Keep the syringe on ice until use; this is intended for injection into the right ventricle to expel blood and halt cardiac contractions.
- Connect the other marked needle to the peristaltic pump tubing designated for heart injections and secure the setup in place (Figure 5D). In this procedure, the tubing is steadily fixed into place using a holder made of P1000 tips taped in a refill wafer. The complete set up of the perfusion system is shown in Figure 5E.

### 4.6. Cardiomyocyte isolation procedure

**Figure 6:**
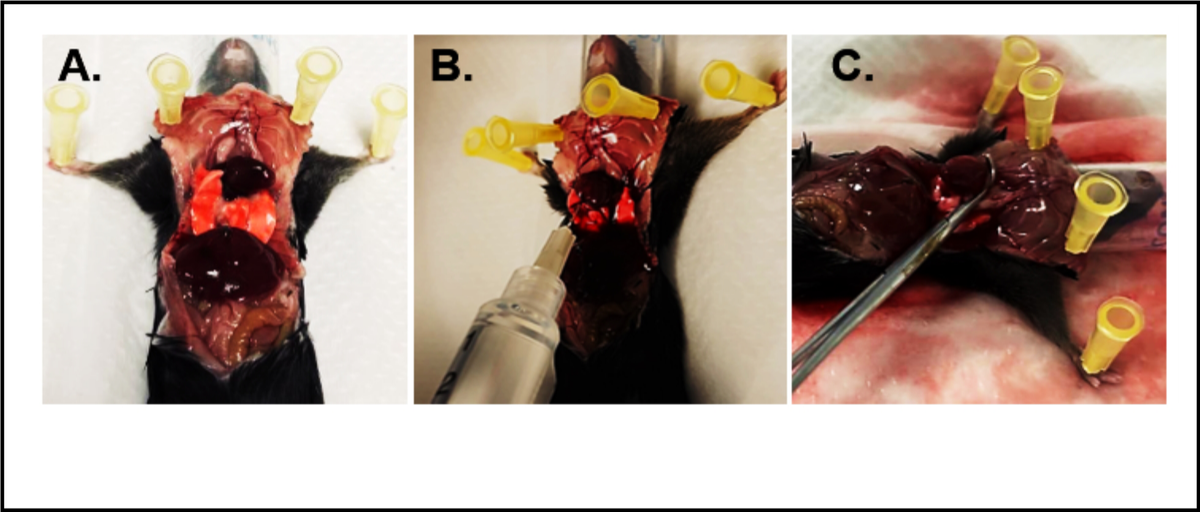
Animal surgical procedure. **A)** Chest cavity opened, exposing the heart; **B)** Injection of EDTA buffer into the right ventricle; **C)** Clamping the aorta

**Figure 7:**
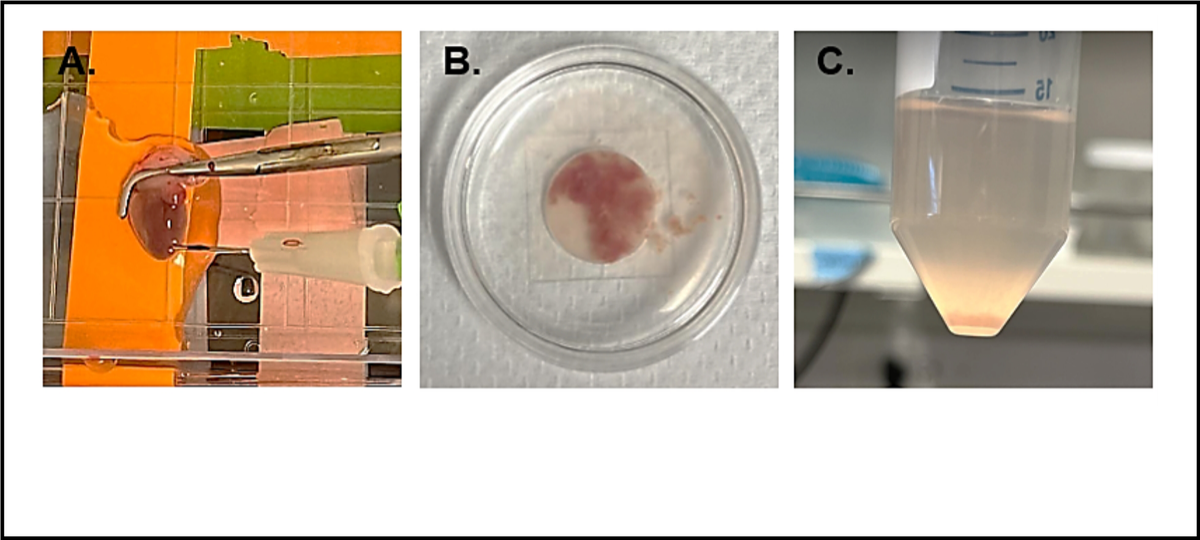
Isolation of cardiomyocytes from the excised heart. **A)** Excised heart connected to the perfusion system via intraventricular injection. **B)** Heart tissue disassociation. **C)**Pellet of healthy cardiomyocytes.

- Anesthetize the mouse with isoflurane and confirm full anesthesia by verifying the absence of a toe-pinch reflex.
- After ensuring the mouse is fully anesthetized, open the chest cavity using sharp scissors. Clean the chest area with 70% ethanol and make an incision below the diaphragm. Locate the septum and gently elevate it to cut laterally below the ribs. Expose the diaphragm by cutting along the rib cage, then carefully slice the ribcage upwards and pin it back to reveal the heart (Figure 6A).
- To clear the heart of blood and cease its contraction, gently move the lungs aside, sever the inferior vena cava and the descending aorta with Vannas Spring Scissors (F.S.T, 91500-09), and inject 5 ml of ice-cold EDTA into the right ventricle using a syringe marked at 3 mm (Figure 6B).
- Clamp the aorta with Micro Hemostatic Forceps 12.5CM, 90 deg ang (WPI, 503360) to secure the heart before excision (Figure 6C).
- Remove the heart and place it in a 100 mm cell culture dish. Insert the 27G needle that is attached to the perfusion system into the heart (Figure 7A). Perfuse the heart with warm EDTA at 1.25 ml/min for 5 minutes, followed by digestion buffer at 1.5 ml/min for 60 minutes.
- Post-digestion, transfer the heart to a 35 mm dish with 1 ml of warm digestion buffer. Mince the tissue thoroughly with sharp tweezers (Figure 7B).
- Move the minced tissue into a 50 ml tube containing 2 ml of warm digestion solution and incubate in a 37°C water bath for 5 minutes. After 5 minutes, pipette the tissue/cell suspension up and down 20 times using a 3 ml transfer pipette. Place the tube in a 37°C water bath for 5 more minutes, then pipette the suspension up and down for another 20 times. At this point, the tissue should be fully digested. Add 5 ml of stop buffer and pipette the suspension up and down for another 5 times.
- Rinse a 100 μm filter 3 times with stop buffer. Transfer the washed 100 μm filter to a new 50 ml conical tube and use a 3 ml transfer pipette to filter the tissue suspension through the filter. Allow the healthy myocytes to settle at room temperature for 15 minutes (Figure 7C).
- Discard the supernatant, resuspend the cardiomyocyte pellet in calcium re-introduction wash buffer 1, and let settle for 15 minutes. Remove the supernatant and repeat the process with calcium re-introduction buffer 2 and buffer 3 sequencially. After the final settling, discard the supernatant, resuspend the cardiomyocyte pellet in 5–8ml of plating media, and seed onto PDL-coated 35 mm glass-bottom dishes. Allow the cells to adhere at room temperature for 20 minutes.

### 4.7. Live cell imaging

#### Preparation of cells:

- After the cells have adhered to the plates, the initial culture medium was replaced with fresh medium with or without LPS (10 ng/ml). Subsequently, the cardiomyocytes were incubated at 37°C for 1 hour.
- After the incubation, the cardiomyocytes were washed once with tyrode buffer and then incubated with MitoTracker Green (100 nM, ThermoFisher Scientific, M7514) together with MitoSOX Red (5 μM, ThermoFisher Scientific, M36008), or with JC-1 (10 μg/ml ThermoFisher Scientific, T3168), in tyrode buffer for 30 minutes at 37°C.

#### Imaging capture:

Post-incubation, the cells were washed twice with tyrode buffer. Imaging was then performed using an LSM 510 confocal microscope, which features an Axio Observer Z1 motorized inverted microscope and Zen software (Carl Zeiss Microscopy), to capture detailed images of the cells.

### 4.8. Transmission Electron Microscopy (TEM) imaging

#### Sample fixation:

- Cardiomyocytes were washed in phosphate-buffered saline (PBS) and then fixed in a solution of 2% paraformaldehyde (Electron Microscopy Sciences), 2.5% glutaraldehyde (Electron Microscopy Sciences), and 0.2% tannic acid (Ted Pella, Inc) in PBS for 1 hour at room temperature.
- After extensive washing with deionized water, the samples were fixed in a solution of 1% osmium tetroxide and 1.5% potassium ferricyanide (Electron Microscopy Sciences) in deionized water for 1 hour at room temperature in the dark.

#### Post-fixation staining:

The samples were en bloc stained with 1% uranyl acetate.

#### Dehydration:

The samples were sequentially dehydrated in an ascending concentration series of alcohols (25%, 50%, 75%, 95%, and 100%, Electron Microscopy Sciences), followed by incubation in propylene oxide.

#### Embedding:

- The samples were embedded in a 1 to 1 ratio of propylene oxide to epoxy resin, comprised of a mixture of EMbed 812, nadic methyl anhydride, dodecenyl succinic anhydride, and 2,4,6-Tris(dimethylaminomethyl)phenol, Electron Microscopy Sciences), for 12 hours on a rotary mixer (Ted Pella, Inc.).

The samples were then fully embedded in 100% epoxy resin for 12 hours at room temperature on the rotary mixer, with a resin change followed by an additional 2-hour incubation. The epoxy resin was allowed to polymerize at 70°C for 36 hours and then allowed to cool to room temperature.

#### Sectioning:

Ultrathin sections (90 nm) were prepared using an ultramicrotome (EM UC7, Leica Microsystems) then mounted on formvar- and carbon-coated 200 mesh copper grids (Electron Microscopy Sciences).

#### Section staining:

Mounted samples were stained with filtered 1% uranyl acetate and Reynold’s lead citrate prior to imaging.

#### Imaging capture:

Imaging was performed using a Philips CM 120 transmission electron microscope (TSS Microscopy) equipped with a BioSprint 16 megapixel digital camera (Advanced Microscopy Techniques).

**Figure 8:**
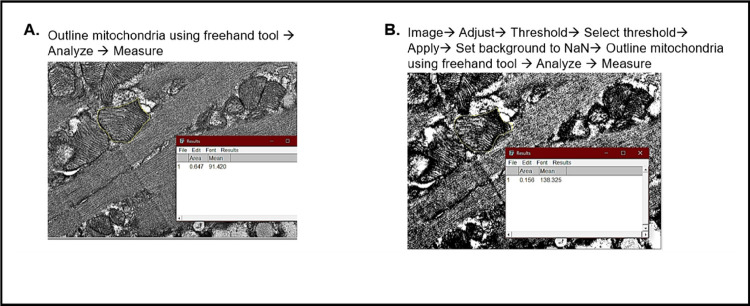
Analysis of mitochondria area and cristae surface area from TEM images. **A)** Calculating mitochondria area. **B)** Calculating area unoccupied by mitochondrial cristae.

Semi-quantification analysis of TEM images: Images were properly scaled and quantitative analysis, including measurements of mitochondria number, area, cristae occupancy, disorganization of cristae, lipid droplet area, and autophagic events, was conducted using ImageJ software. Specifically, mitochondria with normal cristae were characterized by organized, densely packed membranes, whereas those with disorganized cristae were identified by swelling, fragmentation, or irregular shapes. Mitochondrial and lipid droplet areas were measured using the freehand tool (example shown in Figure 8A). Cristae occupancy percentage was calculated by converting images to 32-bit, applying a threshold, and analyzing the outlined area unoccupied by cristae (Figure 8B). The formula used was: Cristae occupancy percentage = (total mitochondrial area − area unoccupied by cristae) / total mitochondrial area × 100.

## Figures and Tables

**Table 1.** 

Compound	Molar Mass g/mol	(mg) to be added	Catalog #
NaCl	58.44	7597.20	Sigma-Aldrich S9625
KCl	74.55	372.75	Sigma-Aldrich P4504
NaH_2_PO_4_.H_2_O	137.99	60.00	Sigma-Aldrich 71496
HEPES	238.30	2383.00	Sigma-Aldrich H4034
Glucose	180.16	1251.50	Sigma-Aldrich G6152
BDM	101.10	1802.60	Sigma-Aldrich B0753
Taurine	125.15	1011.00	Sigma-Aldrich T0625

**Table 2:** 

Enzyme	Final Concentration (mg/ml)	Volume required (ml)
Collagenase 2	0.5	0.5
Collagenase 4	0.5	0.5
Protease XIV	0.05	0.05
Perfusion Buffer		49

**Table 3:** 

Compound	Stock Concentration	Final Concentration	ml/1ml media required
M199 (ThermoFisher Scientific, 11043023)			880
FBS	100%	10%	100
BDM	1M	10 mM	10
P/S	100×	1×	10

* note: To make 1M 2,3-Butanedione monoxime (BDM) solution, dissolve 0.101 g of BDM in 1 ml of distilled water and incubate the solution at 37°C with gentle shaking until BDM is fully dissolved.

**Table 4:** 

Solution	ml for Buffer 1	ml for Buffer 2	ml for Buffer 3
**Perfusion Buffer**	6	4	2
**Plating Media**	2	4	6

## Data Availability

The results and images generated and/or analyzed during the current study are available from the corresponding author on reasonable request.
